# Effect of Through-the-Thickness Delamination Position on the R-Curve Behavior of Plain-Woven ENF Specimens

**DOI:** 10.3390/ma16051811

**Published:** 2023-02-22

**Authors:** Mazaher Salamat-Talab, Alireza Akhavan-Safar, Ali Zeinolabedin-Beygi, Ricardo J. C. Carbas, Lucas F. M. da Silva

**Affiliations:** 1 Department of Mechanical Engineering, Arak University of Technology, Arak 38181-41167, Iran; 2 Institute of Science and Innovation in Mechanical and Industrial Engineering (INEGI), Rua Dr. Roberto Frias 400, 4200-465 Porto, Portugal; 3 Departamento de Engenharia Mecânica, Faculdade de Engenharia, Universidade do Porto, Rua Dr. Roberto Frias, 4200-465 Porto, Portugal

**Keywords:** delamination, plain-woven composites, mode II interlaminar fracture toughness, cohesive zone model (CZM), virtual crack closure technique

## Abstract

In this study, the effect of through-the-thickness delamination plane position on the R-curve behavior of end-notch-flexure (ENF) specimens was investigated using experimental and numerical procedures. From the experimental point of view, plain-woven E-glass/epoxy ENF specimens with two different delamination planes, i.e., [0_12_//0_12_] and [0_17_//0_7_], were manufactured by hand lay-up method. Afterward, fracture tests were conducted on the specimens by aiding ASTM standards. The main three parameters of R-curves, including the initiation and propagation of mode II interlaminar fracture toughness and the fracture process zone length, were analyzed. The experimental results revealed that changing the delamination position in ENF specimen has a negligible effect on the initiation and steady steady-state toughness values of delamination. In the numerical part, the virtual crack closure technique (VCCT) was used in order to analyze the imitation delamination toughness as well as the contribution of another mode on the obtained delamination toughness. The numerical results indicated that by choosing an appropriate value of cohesive parameters, the trilinear cohesive zone model (CZM) is capable of predicting the initiation as well as propagation of the ENF specimens. Finally, the damage mechanisms at the delaminated interface were investigated with microscopic images taken using a scanning electron microscope.

## 1. Introduction

Polymer matrix composites (PMCs) are currently of interest to industry and academia—especially in the automotive, aerospace, wind turbine, military, and aerospace sectors—due to their superior properties, such as high strength-to-weight ratio and good mechanical performance. The use of glass-fiber-reinforced polymer (GFRP) [1,2] and carbon-fiber-reinforced polymer (CFRP) [3] has become more established because of their good mechanical properties. One of the most important mechanisms leading to damage in these materials is delamination. Delamination refers to a crack that propagates between two adjacent layers in laminated composites causing a decrease in the strength and stiffness of the structure, often leading to sudden and catastrophic failures. Investigating the delamination behavior of laminated composites in different failure modes has attracted the attention of many researchers [4,5,6,7,8,9]. One of the key parameters in the delamination analysis of composites is fracture toughness, which has been studied by several researchers [10,11,12,13,14,15,16]. Various samples, such as end-notched flexure (ENF) specimens, 4ENF, end-loaded split (ELS), and over notched flexure (ONF) are used to investigate the mode II interlaminar fracture toughness of laminated composites.

Morais and Pereira [17] investigated the mode II interlaminar fracture in ENF and 4ENF carbon/epoxy samples with unidirectional fibers using the effective crack method. Parameters including the beam thickness, width, crack position, and stacking sequence affect the mode II fracture toughness. Wang et al. [18] investigated the mode II interlaminar fracture toughness using four types of ENF, ELS, 4ENF, and ONF test specimens for unidirectional carbon/epoxy composites. The results showed that the initial fracture toughness value obtained from the ENF test has a smaller scatter in the obtained experimental data. Additionally, the fracture toughness obtained from the ENF test was almost 2% lower than the 4ENF test. Andersons and Konig [19] comprehensively investigated the effect of different parameters on the fracture toughness of laminated composites. They observed that the traditional fracture toughness characterization through various tests on unidirectional composites (according to the interface lay-up and loading mode) can lead to over- or underestimation of crack growth resistance. Yashiro et al. [20] characterized the behavior of the R-curve under mode II loading condition. For this purpose, they used double-end notched tension samples. The results showed that the mode II fracture toughness corresponds well with the results obtained from ENF samples. Chai [21] investigated the effect of the interface fiber angle and the properties of the matrix on the fracture toughness of laminated composites with carbon fibers. However, in this study, he did not report the resistance curve of ENF samples. Hosseini et al. [22] investigated the effect of the mat layer between the woven fiber layers on the mode II fracture toughness of glass/epoxy composites. They showed that the presence of the mat layer between the layers reduces the initiation and propagation values of fracture toughness by 59% and 35%, respectively. Mollón et al. [23] analyzed the effect of crack plane position on the critical strain energy release rate in carbon/epoxy ENF samples considering unidirectional fibers. They found that the deviation of the crack plane from the middle surface of the sample has no effect on the strain energy release rate of mode II. These researchers used a numerical method based on the virtual crack-closure technique (VCCT) to obtain fracture toughness. The effect of interfacial angles 0/θ and θ/−θ in multidirectional samples on the mode II fracture toughness was studied by Pereira et al. [24]. The results showed that increasing the angle θ in the studied delamination interface angles causes an increase in the interlaminar fracture toughness. Akhavan-Safar et al. [25] studied the effect of natural date palm tree fibers on the resistance curve of ENF samples. They showed that adding 5 wt% of petiole fibers increased the initial fracture toughness of glass/epoxy laminated composites by 61%. Additionally, they found that adding 5 wt% of bunch fibers improved the propagation of mode II interlaminar fracture toughness by 73%.

Numerical modeling of the delamination phenomenon can be conducted using two common methods: cohesive zone model (CZM) and VCCT. CZM is a suitable choice for crack initiation and propagation analysis in various materials, such as metals, adhesives, and laminated composites, whose main advantage is the elimination of the singularity at the crack tip.

The traction–separation model is considered as a key parameter for CZM [26,27]. Many researchers have used CZM to numerically simulate the load–displacement behavior of ENF specimens [28,29,30,31,32,33]. Moura and Morais [34] used a bilinear CZM based on 3D interlayer finite element modeling for ENF and ELS samples. Chandra et al. [35] compared the exponential and bilinear CZM shapes. They showed that the shape of the interface laws has a significant effect on the numerical simulation results. Williams and Hadavinia [36] analytically investigated the initiation and propagation of delamination in composites. Anyfantis and Tsouvalis [37] studied the resistance curve behavior of unidirectional ENF samples by using different data reduction methods, including corrected beam theory (CBT), the compliance calibration method (CCM), and the compliance-based beam method (CBBM). In addition, they used different CZM shapes, such as trapezoidal, linear-exponential, and bilinear. They found that the CBBM in relation to the trapezoidal CZM can estimate the experimental data more precisely. In another study, five CZM shapes, including the equivalent linear elastic, equivalent constant stress, cubic, bilinear, and exponential models with the assumption of interface shear strength and mode II fracture toughness, were analyzed by Ouyang et al. [38]. It was found that the CZM shape has a great effect on the critical load and the load–displacement curve for short cracks. Salamat-Talab et al. [39] used bilinear, trapezoidal, exponential, and linear-exponential models to predict the load–displacement behavior in unidirectional laminated composite under mode II loading. The results showed that the use of linear–exponential softening behavior provides a suitable answer for estimating the load-displacement. Heidari-Rarani and Ghasemi [40] developed a trilinear CZM for ENF samples by using the effect of the R-curve. They found the cohesive parameters using a semi-analytical method and showed the effectiveness of their model for analyzing the propagation behavior. Dourado et al. [41] presented a bilinear model for unidirectional specimens with a large fracture process zone. The model parameters were determined by fitting the load-displacement data obtained from finite element analysis (FEA) with the relevant experimental data.

As indicated above, despite the extensive analysis of mode II fracture toughness of composites, however, the effect of through-the-thickness delamination position on the mode II interlaminar fracture toughness and on the cohesive modelling of plain-woven laminated composites has not been well explored. Accordingly, the present study deals with this topic. To achieve this, in the first step, the delamination behavior of symmetric and asymmetric ENF samples manufactured by E-glass/epoxy plain woven laminated composite was experimentally investigated. Then, the distribution of the strain energy release rate across the sample was obtained numerically using VCCT. In addition, the suitable CZM shape for predicting the initiation and propagation of delamination was also investigated and the most accurate cohesive parameters were presented. Numerical and experimental results were also compared.

## 2. Properties and Fabrication of Samples

**Figure 1:**
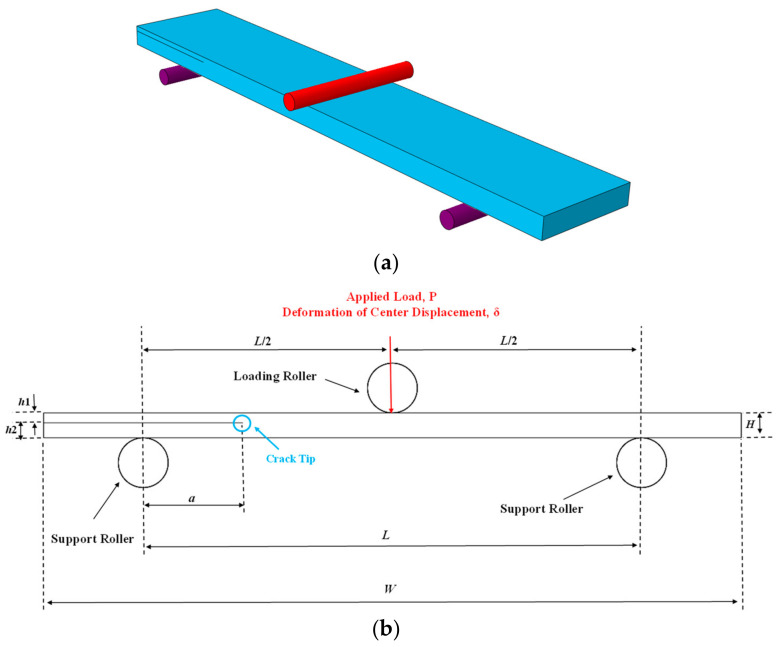
Schematic and geometrical configuration of ENF specimen (**a**) 3D scheme (**b**) dimensions and boundary condition.

The composite samples used in this study consist of 24 layers made using the hand lay-up method. The fibers used are woven glass fibers of 200 g/m^2^ with a harness of 2. EPL 1012 resin and RH 112 hardener with a weight ratio of 100 to 12 were used to make the plain-woven composites. In order to create pre-cracks within the laminates, a Teflon tape with a thickness of 13 microns was used. In addition, to change the position of the crack in the samples, the stacking sequences of [0_12_//0_12_] and [0_17_//0_7_]—where “//” indicates the position of the crack between the layers—were used. In the stacking sequences of [0_12_//0_12_], the crack was placed between Layers 12 and 13, and in the stacking sequences of [0_17_//0_7_], the crack was placed between Layers 17 and 18. Considering that the fabric is used with harness 2, so the layer shown with zero angle has 90-degree fibers equal to zero fibers. The samples were placed at ambient temperature for 24 h for the initial curing, and after that, in order to post-cure, they were subjected to a temperature of 60 °C for 4 h (according to the manufacturer’s recommendation) to improve their mechanical properties. ENF samples were cut to a width of 25 mm and a total length of 140 mm, as shown in Figure 1. The geometrical properties of the symmetric and asymmetric ENFs are summarized in Table 1. After cutting, the edges of the sample were polished with soft sandpaper to better show the crack during growth. It should be noted that for the repeatability of the test results, at least three specimens were manufactured and tested for each stacking sequence. Tensile test samples and three-point bending tests were performed to determine the mechanical properties of plain-woven composites such as in-plane shear modulus and flexural modulus according to ASTM D3518 [42] and ASTM D790 [43] standards, respectively. Additionally, in accordance with the ASTM D3039 [44] standard, the longitudinal and transverse elastic modulus were determined, as shown in Table 2.

### Fracture Test and Data Reduction Method

A universal testing machine (Santam-STM-150, SANTAM Engineering Design Company, Tehran, Iran) and a Bongshin load cell with a capacity of 2000 Kgf were used to conduct the fracture tests and to obtain the load–displacement curves. For this purpose, to determine the mode II fracture toughness according to the ASTM D7905 standard [45], which proposes the compliance calibration approach, three-point bending tests were first carried out on the ENF samples along the cracks of 20, 25, 35, and 40 mm lengths to obtain the compliance of the specimens. It should be noted that the crack length is measured from the center of the left roller support. Then, the fracture tests were conducted on specimens with a crack length of 30 mm. The crosshead speed for the test was considered to be 0.5 mm/min. In addition, based on the above standard, the mode II interlaminar fracture toughness was obtained using Equation (1):

(1) $$G_{IIc}=\frac{3P^{2}ma^{2}}{2b}$$

**Figure 2:**
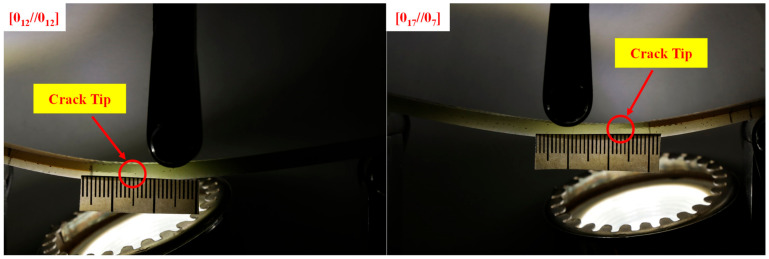
ENF specimens under mode II loading.

In this equation, G*_IIC_* is the mode II interlaminar fracture toughness, *a* is the crack length, m is the calibration parameter, P is the applied load along the crack length, and b is the sample width. To obtain the calibration parameter, it is necessary to draw the compliance diagram in terms of the third power of the crack length. The slope of the curve fitted to this plot indicates the m value. To measure the length of the crack, according to Figure 2, a sticky paper ruler was placed on the side of the samples, and the length of the crack was measured during the test. It should be noted that in order to obtain the interlaminar fracture toughness, the load, the load line displacement, and the length of the crack should be recorded simultaneously. The load and displacement values were recorded at 10 s intervals, and to measure the crack length, images of the sample were taken at the same time interval with a suitable quality (4000 × 6000 pixels) using the Canon D80 digital camera (Canon Inc., Tokyo, Japan).

## 3. Results and Discussion

**Figure 3:**
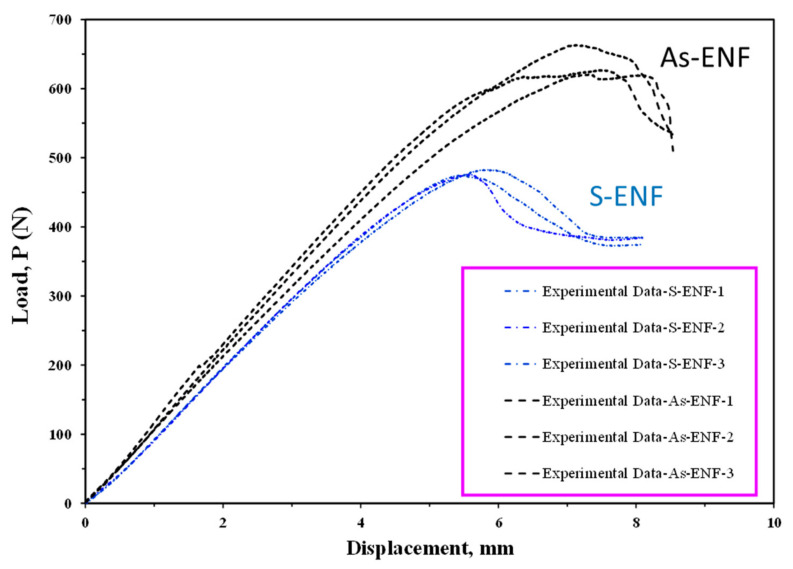
Load–displacement curves of ENF specimens with symmetric (S-ENF) and asymmetric (As-ENF) delamination positions.

The load–displacement curves for the ENF samples obtained from the fracture test are illustrated in Figure 3. The behavior of these graphs is initially linear due to the elastic behavior of these materials, and then it changes to a nonlinear state, which actually indicates initial crack growth in composite samples. These data were used to calculate the initial fracture toughness of the samples. As the graphs become nonlinear, the stiffness decreases but the load continues to increase, and after it has reached its maximum value, the fracture toughness fluctuates around a certain value, which corresponds to the propagation value or steady-state value of fracture toughness. It should be noted that after the load reaches its maximum value, with the increase in the delamination length, the load starts to decrease. According to Figure 3, it is clear that the maximum load in the stacking sequence of [0_17_//0_7_] is higher than that of the [0_12_//0_12_] because of the higher stiffness of samples with the [0_17_//0_7_] stacking sequence [46]. In addition, the behavior of the load–displacement curve indicates that the crack growth in plain-woven composites has occurred in a stable manner. The symmetry (S-ENF) and asymmetry (As-ENF) results in Figure 3 correspond to samples with [0_12_//0_12_] and [0_17_//0_7_] stacking sequence, respectively.

### 3.1. Fracture Toughness and R-Curve

**Figure 4:**
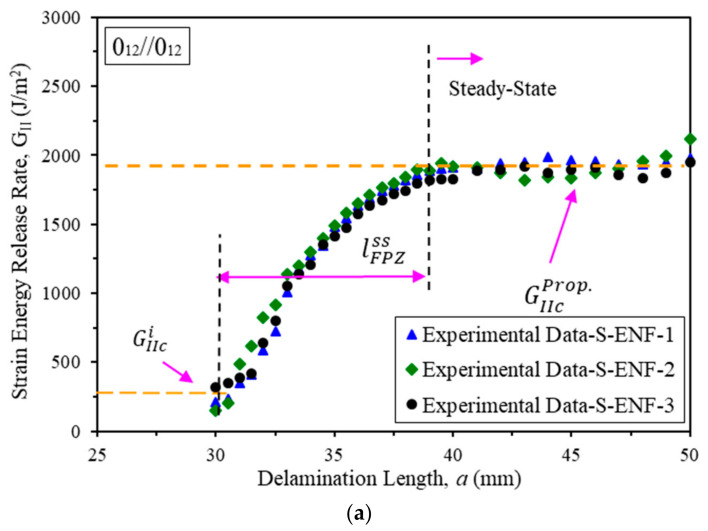

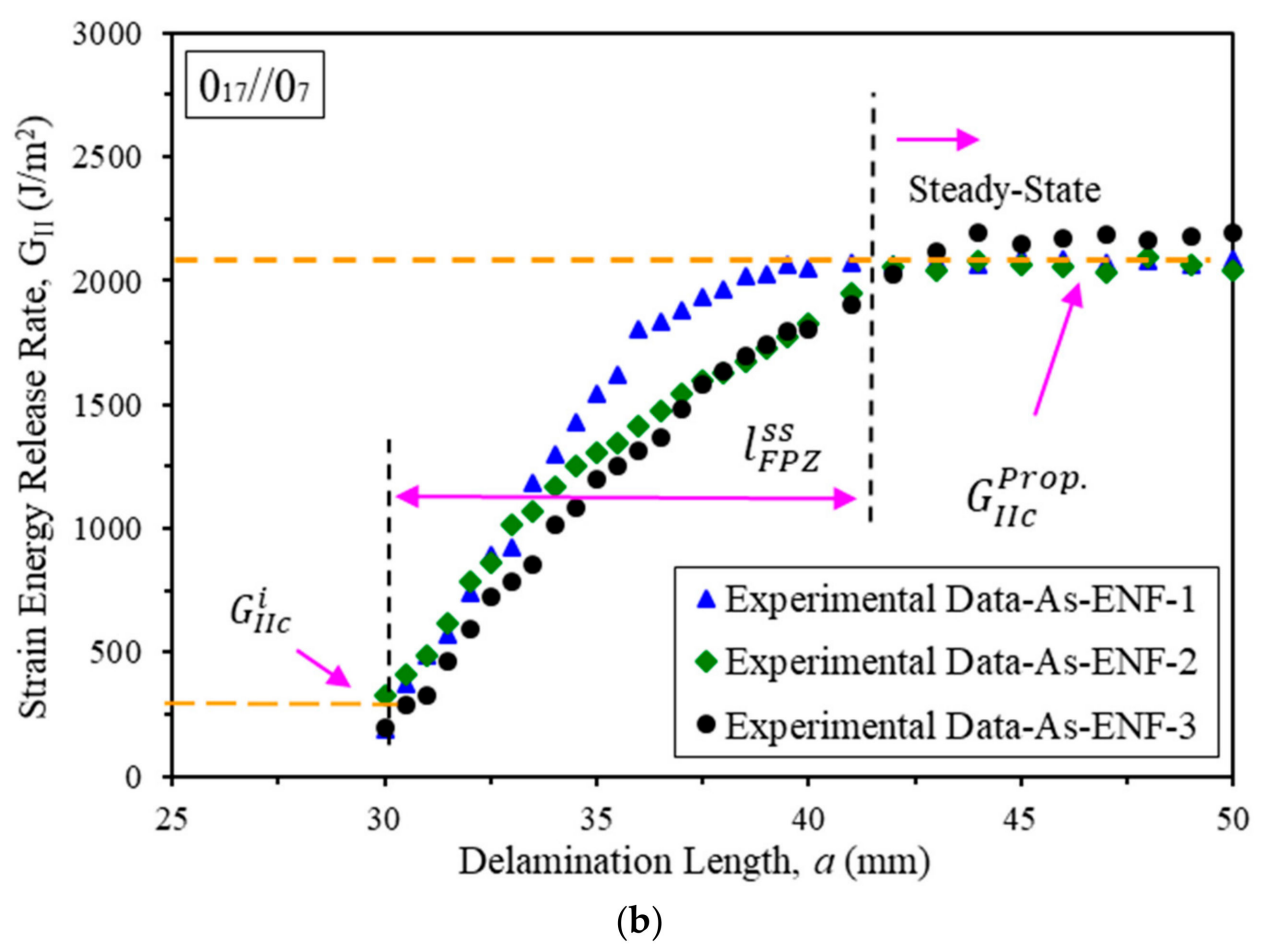
R-curves of ENF specimens with (**a**) symmetric delamination planes (S-ENF) and (**b**) asymmetric delamination planes (As-ENF).

Figure 4 shows the R-curves (resistance curve that denotes the strain energy release rate (*G_II_*) vs. the delamination length) of ENF samples with symmetric and asymmetric delamination planes. Comparing the results for two symmetric and asymmetric samples shows that the initiation and propagation of mode II interlaminar fracture toughness for ENF specimens with asymmetric delamination positions through the thickness increases by 4.85% and 9.28%, respectively, which is not a significant increase. In other words, two samples have almost identical R-curves. Maximum load, fracture process zone length, and initiation and propagation values of fracture toughness are listed in Table 3. It should be mentioned that the length at which the crack starts to grow to reach the steady state fracture toughness is called steady state fracture process zone (FPZ) length, $l_{FPZ}^{ss}$.

### 3.2. Morphology

**Figure 5:**
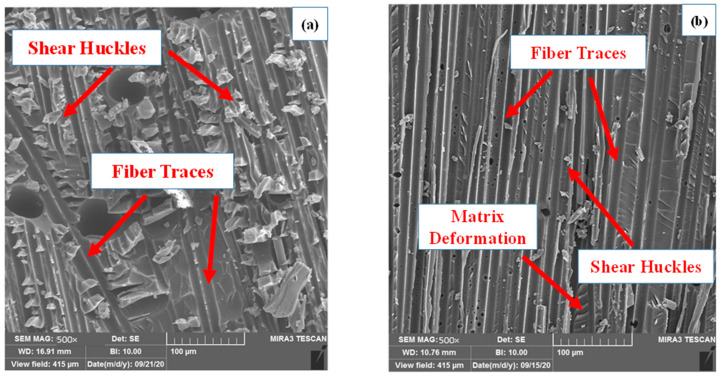
SEM images of delamination interfaces of (**a**) S-ENF and (**b**) As-ENF samples.

In order to better understand the mechanisms of crack initiation and propagation, images of the interface of ENF samples with different delamination planes were prepared by the scanning electron microscope (SEM) method. As a significant result in Figure 5, it can be concluded that the main mechanism of crack growth in both samples is shear huckles, which occur due to shear stresses at the interface. Additionally, fiber traces can be mentioned as a mechanism of crack growth, which shows the effect of fiber residue on the resin. Therefore, since the damage mechanisms at the interface of the samples are similar, there is no significant difference in their fracture toughness.

## 4. Finite Element Simulation of the Delamination Growth

### 4.1. Simulation of the Delamination Using CZM

In this section, the numerical simulation of delamination growth in ENF samples using Abaqus software is discussed. One of the important goals in this study is the evaluation and efficiency of the cohesive model in simulating the fracture process. It should be noted that for the numerical simulation, the dimensions of the samples considered for fracture tests were taken into account.

**Figure 6:**
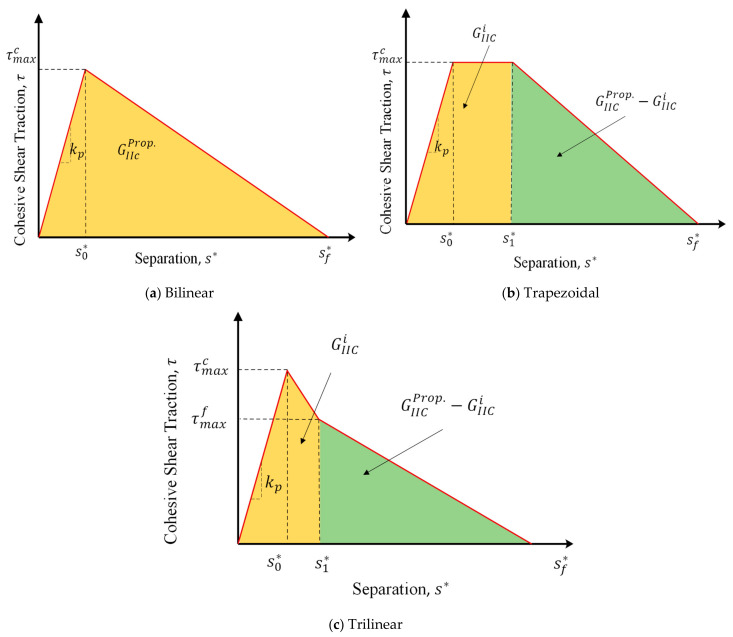
Different types of cohesive shear traction versus separation.

In CZM, the crack growth can be modeled as a gradual decrease in the stiffness of the cohesive element, which is based on the softening of the elements at the crack tip region. When the stress reaches the maximum value, the gradual decrease in the stiffness of the cohesive element begins, and when it reaches zero, the element completely fails. In this research, three different CZMs have been used, as shown in Figure 6a–c. In the bilinear CZM, the stress value increases linearly and after reaching the maximum cohesive shear stress, the damage starts in the element. The softening zone in this type of model is linear as shown in Figure 6a. However, in this model, the process of increasing the damage parameter is nonlinear. In the second model, which is a trapezoidal model, after the initial linear and elastic region, the constant shear stress region is experienced in the cohesive zone. In other words, when the amount of slipping increases in the delamination area, the amount of shear stress is constant, but damage occurs in the element. It should also be noted that the area under the CZM curve up to point $s_{1}^{*}\;$is equal to the initial fracture toughness. After this part, the linear softening area of the cohesive element is created until finally, the tolerable traction value in the element reaches zero. In the third model, which is the trilinear model, after the elastic region, there are two linear softening regions with unequal slopes. The first softening zone continues until the area under the curve in this zone reaches the value of the initial fracture toughness. Then, the softening region continues with another slope and linearly, and the amount of traction reaches zero at the end of the damage. The area under the mentioned curve is also equal to the difference between the initial and propagation fracture toughness. Unlike other models, in the bilinear model, there are not any steps that separates the initiation and propagation areas. Therefore, the entire area under the curve is considered to be propagation. In the following, the performance of each of these models in predicting the delamination behavior of symmetric and asymmetric ENF samples is examined. $k_{p}$ is the stiffness of the linear–elastic region of the traction–separation law. In this study, for interfacial stiffness, values between $10^{4}\frac{N}{{\mathrm{mm}}^{3}}$ to $10^{6}\frac{N}{{\mathrm{mm}}^{3}}$ were used, which has an insignificant effect in the numerical results [39]. The $s_{f}^{*}$ is the final point of damage and after this point, no traction exists between the two crack surfaces. This parameter in cohesive laws can be calculated using the other defined parameters.

**Figure 7:**
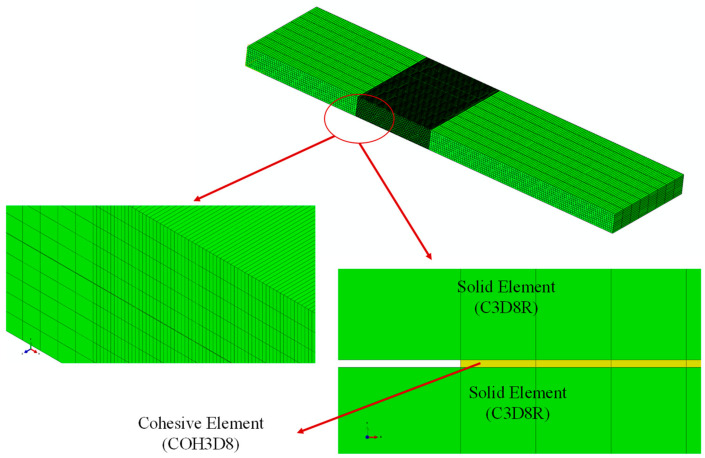
Mesh generated for the ENF specimens.

In order to model the delamination area and the possible path of crack growth, eight-node three-dimensional cohesive (COH3D8) elements with a thickness of 0.01 mm were used. In addition, eight-node linear brick (C3D8R) elements were used for the whole ENF sample. For more accurate numerical results and to prevent divergence of finite element analysis, as well as to shorten the analysis time, in the middle section of the beam along the longitudinal direction, after the crack tip, the mesh was refined (using 10 elements for each 1 mm), while in the other sections 2 elements were used per 1 mm. Additionally, in this section, 10 elements were used along the width of the beam (8 elements per 1 mm), and for the other sections of the beam, 2 elements per 1 mm were used through the longitudinal direction. In the transverse direction, 10 elements are considered (Figure 7). The simply supported boundary conditions are considered for the right and left nodes of the ENF beam, and the vertical displacement is taken for the node regions of the upper arm of the beam at the loading point.

**Figure 8:**
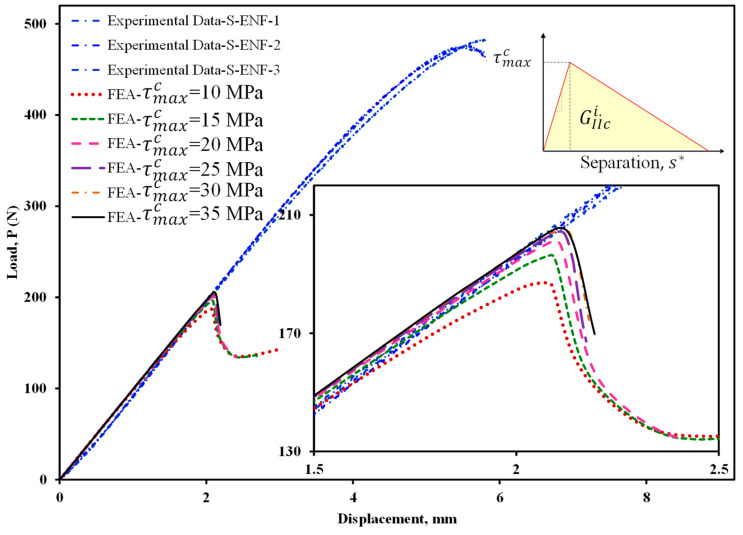
Prediction of initiation point of load–displacement curves by FEA using bilinear CZM and initiation fracture toughness.

**Figure 9:**
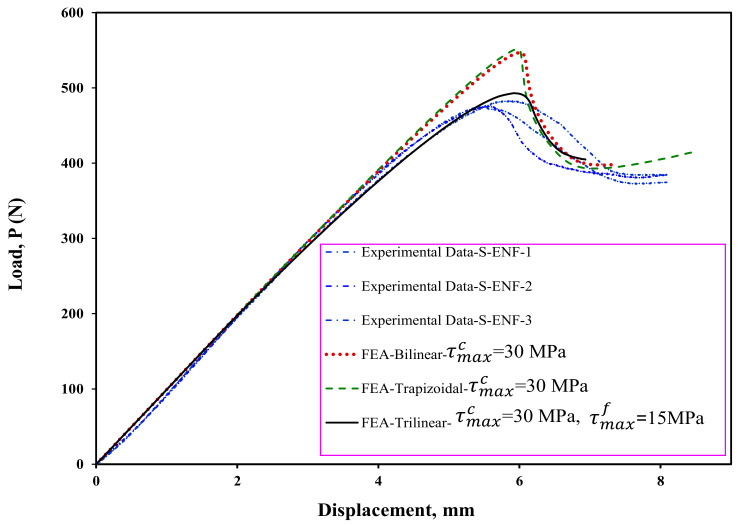
A comparison of the load–displacement curves of experiments and estimates using FEA for ENF specimen with symmetric delamination plane (S-ENF).

**Figure 10:**
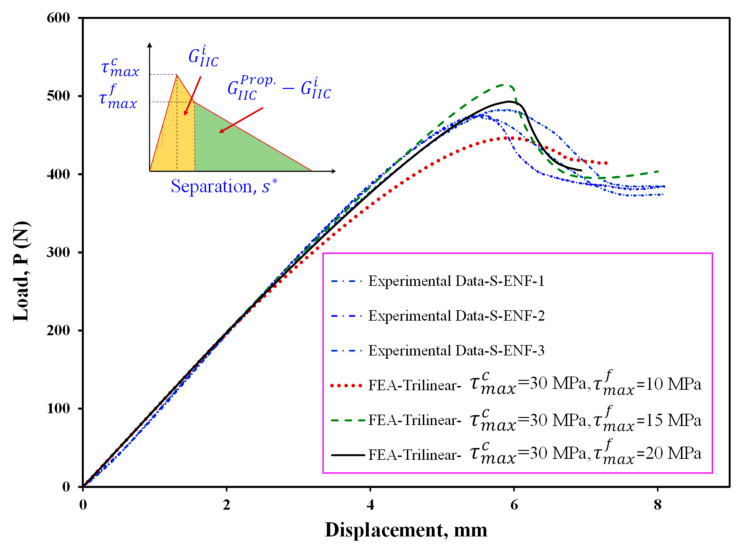
Effect of trilinear CZM parameters on the predicted load–displacement curve for ENF specimen with symmetric delamination plane (S-ENF).

**Figure 11:**
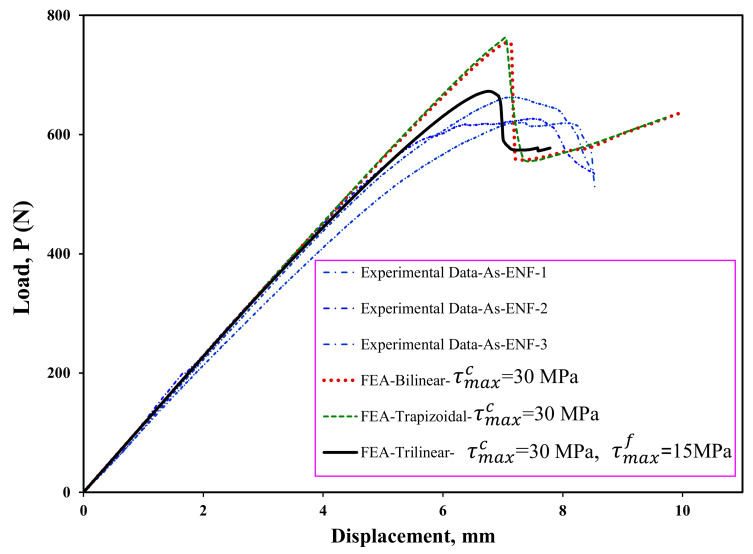
A comparison of the load–displacement curves of experiments and estimated by FEA for ENF specimen with asymmetric delamination plane (As-ENF).

Maximum cohesive shear traction is one of the important and effective parameters in CZM for load–displacement prediction in FEA. In order to determine the value of the maximum cohesive shear traction, first—using the bilinear model and different values of the maximum stress—the starting point of delamination is predicted. By considering the numerical results provided in Figure 8, the stiffness and maximum load increase with increasing the maximum cohesive shear stress, $\tau_{Max}^{c}$. In addition, it can be seen that this increase is stopped when the maximum traction reaches 30 MPa. Therefore, in this study, the maximum stress value in the cohesive area is considered to be 30 MPa in all numerical models. It should be noted that considering the results shown in Figure 8, using the initial fracture toughness the delamination behavior of the symmetric ENF sample cannot be predicted well, and therefore, it is necessary to use the propagation fracture toughness in FEA in order to correctly model the delamination behavior of these samples. In Figure 9, the finite element results are compared to experimental ones. Numerical results show that the bilinear and trapezoidal models are not able to predict the delamination behavior of the sample when the delamination growth starts. Additionally, the predicted maximum load in these CZM types (bilinear and trapezoidal) is much higher than its experimental values. In order to complete and correct these numerical results, a trilinear model has been used to predict the load–displacement curves. As can be seen in Figure 10, the value of $\tau_{Max}^{f}$ has a great influence on the nonlinear region when the delamination growth starts. It also influences the numerically obtained maximum load. Additionally, the maximum load predicted in the finite element model increases with this stress. By using the trial and error method, the value of this stress has been considered to be approximately 15 MPa. The numerical results for this stress indicated a good compliance of the load-displacement curve of the finite element model with its experimental results. Now, according to the stresses obtained, the trilinear model with the obtained stresses was used to model the initiation and propagation of the delamination in the asymmetric ENF samples. The numerical results are compared with the experimental data in Figure 11. Numerical results show that the trilinear model was able to predict more precisely the load–displacement behavior of the asymmetric sample. In other words, it can be said that by extracting the CZM in the symmetric ENF sample, the obtained cohesive model parameters can be used to predict the delamination behavior of the samples that are also asymmetric and under mode II loading.

### 4.2. Effect Delamination Position and Mode Mixity on the Obtained Fracture Toughness

**Figure 12:**
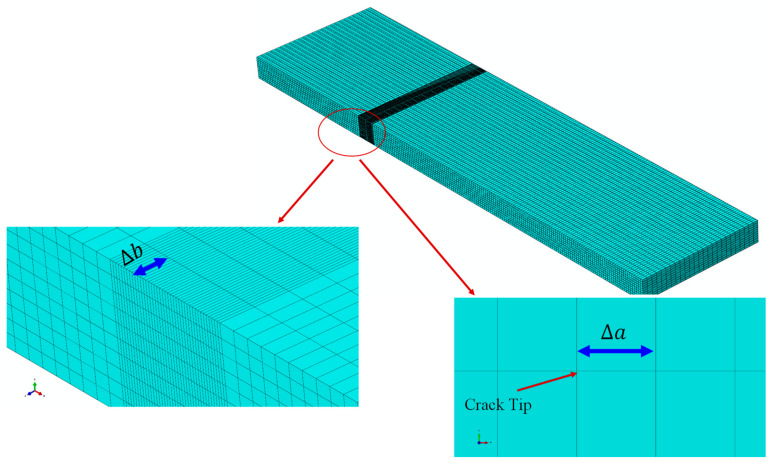
Meshing style of the specimen and the magnified view of the crack tip of ENF specimen.

**Figure 13:**
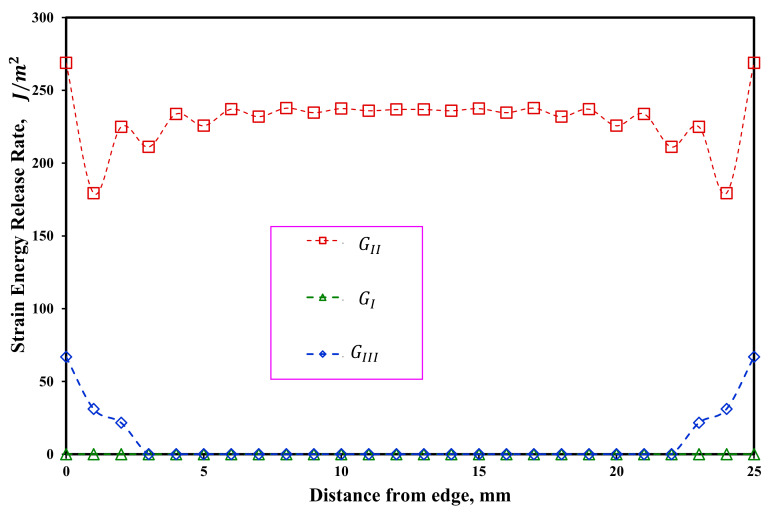
Distribution of the strain energy release rate along the width of ENF specimens with symmetric delamination plane (S-ENF).

**Figure 14:**
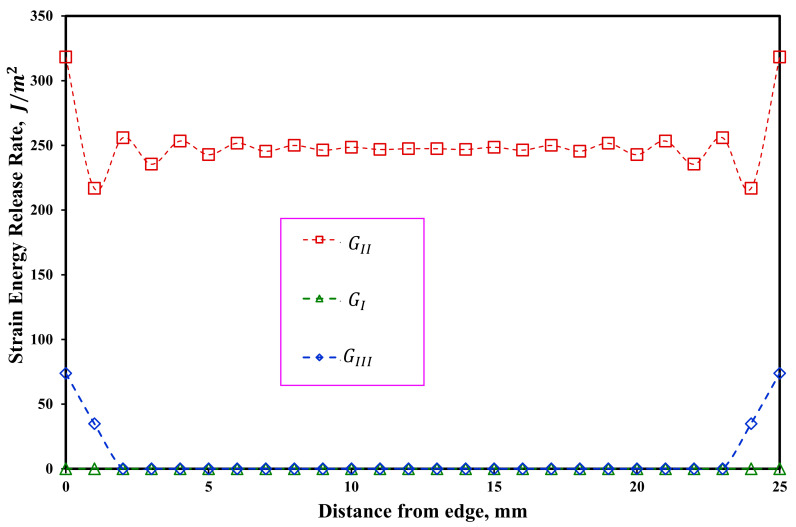
Distribution of the strain energy release rate along the width of ENF specimens with asymmetric delamination plane (As-ENF).

In order to check the distribution of strain energy release rate in symmetric and asymmetric ENF samples, the VCCT method has been used by authors [47,48,49]. In order to numerically model these samples, a three-dimensional model according to Figure 12 was used. In Figure 12, Δa and Δb are the crack extension along the longitudinal direction and width wise of the delaminated beam, respectively. The boundary conditions in this modeling are the same as in the previous case. The strain energy release rate is highly dependent on the considered crack extension value (Δa). In other words, the length of the element considered at the crack tip. In this research, by considering different lengths for the elements near the crack tip, the sensitivity of the results to the size of the elements was investigated. Numerical results showed that the value of Δa = 0.1 mm is a suitable length for the element near the crack tip. It should be also noted that 25 elements were considered in the width of the beam, and 5 elements were considered for each of the arms of the beam along its thickness. The numerical results of the strain energy release rate distribution across the width of the beam for two ENF samples with symmetric and asymmetric delamination planes are shown in Figure 13 and Figure 14, respectively. Accordingly, the mode II strain energy release rate has a uniform distribution along the beam width except in the vicinity of the two edges. In addition, the mode III strain energy release rate contributed to the obtained results near both edges of the ENF beam due to the free edge effect [49]. Additonaly, mode I strain energy release rate is approximately zero along with the beam width. It can be concluded that the mode mixity has a negligible effect on the extracted strain energy release rate. In other words, pure mode II occurs in both symmetric and asymmetric samples. It should be noted that in Figure 13 and Figure 14, $G_{I}$, $G_{II}$, and $G_{III}$ are the strain energy release rates in mode I, mode II, and mode III, respectively.

## 5. Conclusions

In the first part of this study, the plain-woven glass/epoxy ENF samples were fabricated with symmetry and asymmetry using the hand lay-up method. Then, according to the ASTM standards, mode II fracture tests were performed. The delamination behavior of the ENF samples was investigated and the R-curves were extracted. The experimental results showed that the initiation and propagation values of fracture toughness through the thickness delamination position of laminated composites increased by 4.85% and 9.28%, respectively, which is not a significant increase. This means that the symmetry and asymmetry samples have almost the same R-curves. In addition, to better understand the mechanisms of delamination initiation and growth, images of the interface of ENF samples were produced using SEM. Shear huckles and fiber traces can be mentioned as the crack growth mechanism. In the second part of this study, using the finite element method, the delamination growth was modeled. In the numerical analysis, CZM was used to predict the initiation and propagation of delamination. Therefore, three different models—including bilinear, trapezoidal, and trilinear—were used, and the performance of each model was investigated. The numerical results indicated that by choosing an appropriate value for cohesive parameters, the trilinear CZM is capable of predicting the initiation as well as propagation of the delamination phenomenon for both the symmetric and asymmetric ENF specimens. In addition, the distribution of strain energy release rate obtained by the VCCT across the beam width showed that due to the free edge effect, the mode III strain energy release rate contributes only in the vicinity of two edges of the beam width. In other words, the contribution of mode III to the obtained results is negligible.

## Figures and Tables

**Table 1:** Geometrical properties of ENF specimen with symmetrical and asymmetrical delamination plane.

Geometrical Parameters	ENF Specimen with Symmetrical Delamination Plane (S-ENF) [0_12_//0_12_]	ENF Specimen with Asymmetrical Delamination Plane (As-ENF) [0_17_//0_7_]
Total length of the beam, *W* (mm)	140	140
Span length, *L* (mm)	100	100
Beam width, *b* (mm)	25	25
Initial crack length, *a* (mm)	30	30
The thickness of arm-1, *h*_1_ (mm)	2.06	1.22
The thickness of arm-2, *h*_2_ (mm)	2.06	2.94
Total thickness of the beam, *H* (mm)	4.12	4.16

**Table 2:** The mechanical properties of E-glass/epoxy laminated composites.

Properties	Value
Longitudinal elastic modulus (E_1_)	19.5 GPa
Transverse elastic modulus (E_2_)	19.5 GPa
In-plane shear modulus (G_12_)	3.1 GPa
Flexural modulus (E_b_)	18.1 GPa
Poisson’s ratio (ν_12_)	0.29

**Table 3:** Average values of maximum load, steady-state FPZ length, initiation, and propagation values fracture toughness for ENF specimens with different delamination planes.

Delamination Plane	Maximum Load, P (N)	Initial Fracture Toughness, $G_{IIC}^{i}$ (J/m^2^)	Propagation Fracture Toughness, $G_{IIC}^{Prop.}$ (J/m^2^)	FPZ Length, $L_{FPZ}^{ss}$ (mm)
[0_12_//0_12_]	477.60 ± 4.48	226.82 ± 85.96	1905.85 ± 34.74	8.67 ± 0.29
[0_17_//0_7_]	636.87 ± 23.03	237.83 ± 77.50	2082.71 ± 63.96	11.50 ± 1.80

## Data Availability

Not applicable.
